# Relation between Red Cell Distribution Width and Mortality in Critically Ill Patients with Acute Respiratory Distress Syndrome

**DOI:** 10.1155/2019/1942078

**Published:** 2019-03-21

**Authors:** Benji Wang, Yuqiang Gong, Binyu Ying, Bihuan Cheng

**Affiliations:** Department of Anesthesiology, Critical Care and Pain Medicine, The Second Affiliated Hospital and Yuying Children's Hospital of Wenzhou Medical University, Wenzhou 325000, Zhejiang, China

## Abstract

**Background:**

Currently, evidence regarding the predictive significance of red blood cell distribution width (RDW) among patients with acute respiratory distress syndrome (ARDS) remains scarce. The aim of this study was to determine the prognostic value of RDW for critically ill patients with ARDS.

**Methods:**

We studied all patients with ARDS from the Multiparameter Intelligent Monitoring in Intensive Care Database III (MIMIC-III) for whom RDW was available. The clinical outcomes were 30-day and 90-day mortality. Analyses included logistic multivariate regression model, Receiver Operating Characteristic (ROC) analysis, and subgroup analysis.

**Results:**

A total of 404 eligible ARDS patients were included. After adjustment for several clinical characteristics related to 30-day mortality, the adjusted OR (95% CIs) for RDW levels ≥14.5% was 1.91 (1.08, 3.39). A similar trend was observed for 90-day mortality. The RDW levels ≥14.5% were also an independent predictor of 90-day mortality (OR, 2.56; 95% CI, 1.50 to 4.37;* P* = 0.0006) compared with the low RDW levels (<14.5%). In subgroup analyses, RDW showed no significant interactions with other relevant risk factors for 30-day mortality.

**Conclusions:**

RDW appeared to be a novel, independent predictor of mortality in critically ill patients with ARDS.

## 1. Introduction

The acute respiratory distress syndrome (ARDS) is a serious complication with high morbidity and mortality [1, 2]. Even with protective lung ventilation or intravenous steroids, many patients remain affected by severe respiratory failure [3, 4]. Considering the severity of ARDS and its poor prognosis in recent years, various studies have been committed to seeking clinical predictors of mortality in ARDS [5–7]. Though multiple predictors have been reported, their predictive power remains controversial [8–13]. Hence, new predictors with stronger predictive power should be sought.

Red blood cell distribution width (RDW) is a measure of the range of variation of circulating erythrocytes and is reported as part of a standard complete blood count [14]. It is used primarily to differentiate among causes of anemia [15]. Previous studies found that RDW was mainly used as a prognostic indicator of various cardiovascular diseases, in addition to anemia [16, 17]. Furthermore, recent studies suggested independent associations between RDW and the risk of several adverse outcomes, including pulmonary hypertension [18], tumor [19], acute kidney injury (AKI) [20], and others [21, 22]. In addition, increased RDW was an independent risk factor for increased mortality in critical illness [23, 24].

To our knowledge, no epidemiological studies have investigated the impact of RDW on prognosis of patients with ARDS. It remains unclear as to whether RDW is a risk factor for ARDS in critical illness. The aim of this study was to determine the prognostic value of RDW for critically ill patients with ARDS.

## 2. Methods

### 2.1. Data Source

The Multiparameter Intelligent Monitoring in Intensive Care Database III version 1.3 (MIMIC-III v1.3) includes more than 40,000 intensive care unit (ICU) patients treated in a variety of critical care units (medical, surgical, coronary care, and neonatal) at Beth Israel Deaconess Medical Center (Boston, MA, USA) from 2001 to 2012 [25]. Our access to the database was approved by the institutional review boards of the Massachusetts Institute of Technology and Beth Israel Deaconess Medical Center after we completed the National Institutes of Health's web-based course and passed the Protecting Human Research Participants exam (No. 6182750). Similar to our previous work [26], we extracted clinical data, including patient demographics and laboratory test results. To protect privacy, the information of included patients was hidden.

### 2.2. Population Selection Criteria

We restricted the search to adult patients (aged 18 years or above) with ARDS using International Classification of Diseases- (ICD-) 9 code (code = “51882” or code = “5185”). Patients with the following criteria were excluded: (1) no RDW measured during ICU stay; (2) hematologic disease such as leukemia and myelodysplastic syndrome; and (3) missing >5% individual data.

### 2.3. Data Extraction

PostgreSQL tool (version 9.6) was used to extract data from MIMIC-III. The data extraction included clinical parameters, laboratory parameters, demographic parameters, and scoring systems. The following comorbidities were extracted: coronary artery disease (CAD), congestive heart failure (CHF), atrial fibrillation (AF), stroke, AKI, pneumonia, liver disease, and chronic obstructive pulmonary disease (COPD). Laboratory measurements included bicarbonate, creatinine, glucose, white blood cells (WBC), hematocrit, hemoglobin, chloride, platelets, sodium, chloride, potassium, blood urea nitrogen (BUN), anion gap, prothrombin time (PT), activated partial thromboplastin time (APTT), and international normalized ratio (INR). Sequential organ failure assessment (SOFA) and the simplified acute physiology score II (SAPS II) were also extracted. Baseline data were extracted within 24 hours after ICU admission. 30-day and 90-day mortality were the clinical endpoints. Data regarding patient death was obtained from Social Security Death Index.

### 2.4. Statistical Analysis

Baseline characteristics of all patients were stratified by RDW tertiles. Continuous variables were expressed as mean ± standard deviation (SD) and categorical variables were expressed as percentage. 95% confidence intervals (CIs) were provided where appropriate. We used the chi-square test for categorical variables and analysis of variance or Kruskal-Wallis test for continuous variables to compare the groups. We used Receiver Operating Characteristic (ROC) to find the best cutoff of RDW and then used logistic regression to determine whether the RDW was independently associated with 30-day and 90-day mortality, with results presented as odds ratios (ORs) with 95% CIs.

We ran two models for each endpoint in our multivariable analyses. Variables based on epidemiological and biological background were incorporated as confounders, and these confounders resulted in a 10% or greater change in the associations with the 30-day and 90-day mortality of RDW [27]. The lower-limit group was considered the control group. In model I, covariates were adjusted only for age, ethnicity, and gender. In model II, covariates were adjusted for age, ethnicity and gender, liver, stroke, SAPSII, SOFA, anion gap, bicarbonate, creatinine, hemoglobin, glucose, CAD, platelets, AF, and CHF. We generated ROC curves to measure the sensitivity and specificity of RDW and calculated the area under the curve (AUC) to ascertain the quality of RDW as a predictor of mortality. Moreover, we determine the relationships between RDW and the classic scoring systems (SOFA scores and SAPSII scores).

We conducted stratification analyses to investigate whether the effect of RDW differed across various subgroups, including gender, ethnicity, CHF, CAD, AF, stroke, COPD, pneumonia, malignancy, AKI, and renal replacement therapy. The data were analyzed using the EmpowerStats version 2.17.8 (http://www.empowerstats.com/cn/) and R software version 3.42.* P* < 0.05 was considered statistically significant and all reported* P* values were two-sided.

## 3. Results

### 3.1. Subject Characteristics

A total of 404 patients who met the inclusion criteria were divided into separate groups according to RDW. There were three equal groups: 124 patients were in the 11.4-13.8% group, 140 patients were in the 13.9-15.3% group, and 140 patients were in the 15.4-29% group. Characteristics and hematologic laboratory data of the study are displayed in Table 1. Patients with a high RDW (RDW > 13.8%) were more likely to be elderly and have lower diastolic blood pressure, mean blood pressure, hematocrit, and hemoglobin than were patients with a low RDW value (RDW ≤ 13.8%). Patients with a high RDW also had higher creatinine, BUN, and PT than did patients with a low RDW. SOFA and SAPSII scores were significantly lower in the low RDW group. Moreover, high RDW was more frequent in patients with CHF, AKI, and liver disease.

### 3.2. Association between RDW and Clinical Endpoints

Logistic regression analysis was used and patients were divided according to the ROC analysis. The best cutoff of RDW was 14.5%. In model I, OR (95% CI) of RDW ≥14.5% compared to the reference levels (<14.5%) was 2.05 (1.25, 3.35) for 30-day mortality after adjustments for age, ethnicity, and gender. This suggested that the increase in RDW was associated with an increase in mortality. Moreover, after adjustment for age, ethnicity and gender, liver, stroke, SAPSII, SOFA, anion gap, bicarbonate, creatinine, hemoglobin, glucose, CAD, platelet, CHF, and AF, the RDW (≥14.5%) was also associated with an increased risk of mortality (adjusted OR, 95% CI: 1.91, 1.08–3.39). There was a similar trend in 90-day mortality. The high RDW (≥14.5%) was also an independent predictor of 90-day mortality after adjustment for potential confounders (adjusted OR, 95% CI: 2.56, 1.50–4.37) (Table 2).

### 3.3. Prediction of Mortality

**Figure 1:**
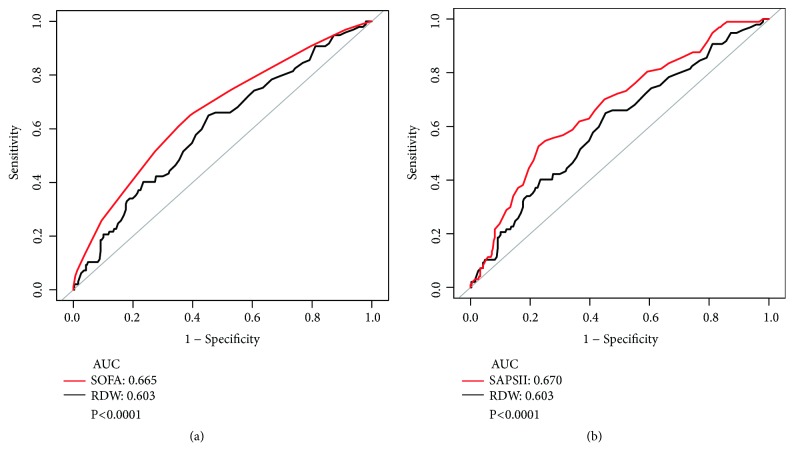
ROC curves for the prediction of mortality in critically ill patients with ARDS. (a) The ability of SOFA scores and RDW scores to predict 30-day mortality. (b) The ability of SAPSII scores and RDW scores to predict 30-day mortality.

ROC curves generated using the indicated variables (RDW, SOFA scores, and SAPSII scores) to predict 30-day mortality were plotted in Figure 1. The AUCs for RDW, SOFA scores, and SAPSII scores were 0.603, 0.665, and 0.670, respectively. The AUC of the RDW was lower than these classical scoring systems (*P* < 0.0001).

### 3.4. Subgroup Analyses

We included gender, ethnicity, CHF, CAD, AF, stroke, COPD, pneumonia, malignancy, AKI, and renal replacement therapy in subgroup analyses (Table 3). The association between the RDW and the risk of 30-day mortality was similar for these strata. No significant interactions were observed (*P* = 0.0591 - 0.8673). RDW showed no significant interactions with any of the other risk factors for 30-day mortality of ARDS.

## 4. Discussion

We found that RDW was an independent predictor of all-cause mortality in patients with ARDS. In addition, RDW showed no significant interactions with any of the other risk factors for 30-day mortality. Felker et al. [28] first investigated the correlation between RDW and the morbidity and mortality of congestive heart failure. Subsequent studies demonstrated that RDW was a novel predictor of multiple adverse outcomes [9, 16, 18, 23]. In contrast to these results, our study also showed a positive correlation between RDW and the mortality of ARDS. Although the AUC of the RDW was lower than SOFA scores and SAPSII scores, it had certain predictive performance. Moreover, there were no significant interactions between RDW and other relevant risk factors, which indicated that no other factors had been identified to influence the association between RDW and all-cause mortality in patients with ARDS

ARDS is a serious life-threatening complication with a high mortality rate [29]. The high mortality appears to be intimately linked to multiple-organ dysfunction syndrome [30]. Diverse biomarkers had been used to predict the prognosis of ARDS in several studies [31–33]. However, most of the studies on biomarkers and ARDS have employed a single pathway approach, though a single mechanism is unlikely to predict the outcome in a syndrome as complex as ARDS [32–34]. RDW is used to measure the variation in circulating erythrocyte volume; it has been extensively investigated in nonhematologic disorders, including autoimmune diseases [35], stroke [36], liver diseases [37], cardiovascular diseases [38], critical illness [39], and respiratory diseases [40]. Therefore, RDW has been regarded as a useful predictive index for numerous diseases and organ dysfunctions. As our study demonstrated, RDW was an independent predictor of mortality in patients with ARDS. It is also an easily available biomarker.

ARDS is a systemic inflammatory response syndrome [41]. The systemic inflammatory response probably helps to explain the potential link between RDW and ARDS; however, the pathophysiologic mechanisms for their relationship remain unknown. Several studies [42, 43] have suggested that increased production of proinflammatory cytokines, neutrophil accumulation, and disruption of pulmonary endothelial and epithelial cell capillary barriers may be the mechanisms of ARDS. Inflammatory reactions affected bone marrow function, and inflammatory cytokines inhibited erythrocyte maturation, resulting in large production of newer reticulocytes, related to the increase in RDW [44]. Moreover, the increase of oxidative stress increased RDW by reducing the survival rate of erythrocytes and by releasing large premature red blood cell into the circulation [45].

Our study had several strengths. First, to the best of our knowledge, this is the first study to comprehensively determine the relationship between RDW and all-cause mortality of ARDS. Furthermore, we selected mortality as the primary outcome.

Our study also had some limitations. First, the study had a single-center retrospective design. It was therefore subject to selection bias; a prospective multicenter study is needed. Second, we measured RDW in patients only upon admission to the ICU and did not assess changes during the ICU stay. Third, as a retrospective study, the number of patients included is not large, and there are many uncertainties in extending this conclusion to other populations, such as different regions, population characteristics, and disease distribution. Fourth, we did not know the patient's serum iron levels or whether erythropoietin use may have affected RDW values. Fifth, some confounders such as previous hemoglobin levels are not available in the MIMIC III; this may affect the reliability of the conclusion. Finally, the limitations of the MIMIC database are inevitable. No mechanism is postulated in our study because it is difficult to study. Moreover, the database contains many inaccurate data elements; these may led clinicians to make incorrect decisions to carry out certain tests and interventions. Therefore, further studies, especially multicenter registry, large-scale, prospective studies, are needed to confirm these findings.

## 5. Conclusions

RDW appeared to be a novel, independent predictor of mortality in critically ill patients with ARDS. Further studies, especially large prospective studies, are needed to confirm the relationship between RDW and adverse clinical outcomes.

## Figures and Tables

**Table 1:** Characteristics of the study patients according to RDW.

Characteristics	RDW,%			*P *value
	11.4 - 13.8	13.9 - 15.3	15.4 - 29	
Clinical parameters, n (%)	124	140	140	
Age, years	59.88 ± 18.52	65.48 ± 16.56	63.42 ± 16.67	0.031
Gender, n (%)				0.624
Female	60 (48.39)	65 (46.43)	73 (52.14)	
Male	68 (54.84)	80 (57.14)	64 (45.71)	
Ethnicity, n (%)				0.266
White	86 (69.35)	107 (76.43)	97 (69.29)	
Black	10 ( 8.06)	9 ( 6.43)	18 (12.86)	
Other	28 (22.58)	24 (17.14)	25 (17.86)	
ICU stay, days	10.23 ± 9.85	8.98 ± 7.02	8.63 ± 9.45	0.307
SBP, mmHg	121.86 ± 18.38	119.16 ± 19.47	116.69 ± 18.44	0.085
DBP, mmHg	63.54 ± 11.33	60.51 ± 11.73	57.30 ± 11.36	<0.001
MBP, mmHg	81.90 ± 11.62	78.88 ± 12.48	75.89 ± 13.08	<0.001
Heart rate, beats/minute	88.86 ± 18.67	91.70 ± 17.23	90.77 ± 17.70	0.426
Respiratory rate, beats/minute	21.29 ± 5.21	21.42 ± 5.12	21.41 ± 4.72	0.970
Temperature, °C	37.12 ± 0.73	37.08 ± 0.70	36.86 ± 0.75	0.006
SPO2_,_ %	96.95 ± 2.28	96.47 ± 2.03	96.57 ± 2.27	0.176
Comorbidities, n (%)				
Coronary artery disease	14 (11.29)	29 (20.71)	24 (17.14)	0.118
Congestive heart failure	9 (7.26)	21 (15.00)	26 (18.57)	0.026
Atrial fibrillation	31 (25.00)	34 (24.29)	40 (28.57)	0.684
Stroke	19 (15.32)	16 (11.43)	11 (7.86)	0.163
AKI	0 (0.00)	11 (7.86)	26 (18.57)	<0.001
Liver disease	5 (4.03)	2 (1.43)	19 (13.57)	<0.001
Pneumonia	68 (54.84)	80 (57.14)	64 (45.71)	0.131
COPD	5 (4.03)	7 (5.00)	4 (2.86)	0.655
Renal replacement therapy	4 (3.23)	14 (10.00)	26 (18.57)	<0.001
Laboratory parameters				
Bicarbonate, mmol/L	24.60 ± 4.20	25.65 ± 4.77	24.19 ± 4.90	0.027
Creatinine, mEq/L	1.35 ± 1.28	1.50 ± 1.38	2.10 ± 1.76	<0.001
Glucose, mg/dl	194.54 ± 117.55	183.37 ± 87.12	184.79 ± 95.91	0.623
WBC, 10^ 9^/L	14.83 ± 6.26	15.94 ± 8.65	19.39 ± 45.41	0.365
Hematocrit, %	38.02 ± 5.36	35.73 ± 5.68	32.14 ± 5.52	<0.001
Hemoglobin, g/dl	12.92 ± 1.88	11.94 ± 1.92	10.60 ± 1.88	<0.001
Platelet, 10^ 9^/L	261.56 ± 99.36	291.76 ± 145.93	227.00 ± 155.77	<0.001
Sodium, mmol/L	140.27 ± 5.46	140.91 ± 4.97	140.16 ± 5.59	0.444
Chloride, mmol/L	107.09 ± 7.06	107.36 ± 6.65	106.07 ± 6.90	0.256
Potassium, mmol/L	4.69 ± 1.54	4.65 ± 0.85	4.68 ± 0.91	0.953
BUN, mg/dl	24.93 ± 17.93	31.09 ± 21.45	42.66 ± 29.72	<0.001
Aniongap, mmol/L	16.53 ± 4.36	16.09 ± 3.55	17.76 ± 5.37	0.006
PT, second	15.28 ± 5.81	15.61 ± 3.82	18.66 ± 7.87	<0.001
APTT, second	43.74 ± 32.96	45.18 ± 32.21	47.46 ± 29.95	0.654
INR	1.64 ± 2.33	1.58 ± 0.82	1.98 ± 1.27	0.093
Scoring systems				
SOFA	4.85 ± 3.26	5.04 ± 3.12	6.34 ± 4.05	<0.001
SAPSII	37.27 ± 13.85	40.16 ± 14.21	43.56 ± 15.69	0.002
30-day mortality, n (%)	21 (16.94)	34 (24.29)	42 (30.00)	0.046
90-day mortality, n (%)	27 (21.77)	43 (30.71)	58 (41.43)	0.003

ICU: intensive care unit; SBP: systolic blood pressure; DBP: diastolic blood pressure; MBP: mean blood pressure; SPO2: blood oxygen saturation; AKI: acute kidney injury; COPD: chronic obstructive pulmonary disease; WBC: white blood cell; BUN: blood urea nitrogen; PT: prothrombin time; APTT: activated partial thromboplastin time; INR: international normalized ratio; SOFA: sequential organ failure assessment; SAPSII: simplified acute physiology score II.

**Table 2:** ORs (95% CIs) for all-cause mortality across groups of RDW.

RDW, %	Non-adjusted		Model I		Model II	
	OR (95%CIs)	*P* value	OR (95%CIs)	*P* value	OR (95%CIs)	*P* value
30-day mortality						
Fitted groups						
<14.5	1.0(ref)		1.0(ref)		1.0(ref)	
≥14.5	2.14 (1.33, 3.44)	0.0017	2.05 (1.25, 3.35)	0.0042	1.91 (1.08, 3.39)	0.0260
90-day mortality						
Fitted groups						
<14.5	1.0(ref)		1.0(ref)		1.0(ref)	
≥14.5	2.51 (1.62, 3.89)	<0.0001	2.48 (1.57, 3.92)	0.0001	2.56 (1.50, 4.37)	0.0006

OR: odds ratio; CI: confidence interval.

Models were derived from logistic regression.

Adjust I model adjust for: age, ethnicity, and gender.

Adjust II model adjust for: age, ethnicity and gender, liver disease, stroke, SAPSII, SOFA, anion gap, bicarbonate, creatinine, hemoglobin, glucose, CAD, platelet, CHF, and atrial fibrillation.

**Table 3:** Subgroup analysis of the associations between RDW and 30-day all-cause mortality.

	No. of patients	OR (95%CI)	*P* value	*P* interaction
Gender				0.4396
F	198	1.64 (0.84, 3.21)	0.1493	
M	206	2.77 (1.40, 5.46)	0.0034	
Ethnicity				0.3069
White	290	2.84 (1.57, 5.14)	0.0006	
Black	37	0.75 (0.14, 3.94)	0.7340	
Other	77	1.40 (0.52, 3.74)	0.5019	
CHF				0.8673
No	348	2.33 (1.41, 3.85)	0.0010	
Yes	56	1.36 (0.30, 6.10)	0.6905	
CAD				0.2028
No	337	2.59 (1.51, 4.44)	0.0005	
Yes	67	0.96 (0.33, 2.83)	0.9410	
AF				0.3863
No	299	2.56 (1.39, 4.72)	0.0026	
Yes	105	1.51 (0.67, 3.37)	0.3193	
Stroke				0.4677
No	358	2.31 (1.37, 3.88)	0.0017	
Yes	46	1.67 (0.47, 5.93)	0.4300	
COPD				0.8542
No	388	2.29 (1.40, 3.75)	0.0009	
Yes	16	0.47 (0.04, 5.90)	0.5561	
Pneumonia				0.4827
No	192	2.63 (1.28, 5.42)	0.0085	
Yes	212	1.81 (0.96, 3.44)	0.0680	
Malignancy				0.7659
No	325	1.75 (1.03, 2.96)	0.0375	
Yes	79	5.81 (1.23, 27.36)	0.0262	
AKI				0.7585
No	367	2.28 (1.37, 3.77)	0.0014	
Yes	37	0.48 (0.08, 2.79)	0.4110	
RRT				0.0591
No	360	2.06 (1.23, 3.45)	0.0059	
Yes	44	1.18 (0.28, 4.97)	0.8174	

CHF: congestive heart failure; CAD: coronary artery disease; AFIB: atrial fibrillation; COPD: chronic obstructive pulmonary disease; AKI: acute kidney injury; RRT: renal replacement therapy.

ORs (95% CIs) were derived from logistic multivariate regression models.

## Data Availability

The clinical data used to support the findings of this study were supplied by Monitoring in Intensive Care Database III version 1.3 (MIMIC-III v.1.3). Although the database is publicly and freely available, researchers must complete the National Institutes of Health's web-based course known as Protecting Human Research Participants to apply for permission to access the database.

## References

[1] Rubenfeld G. D., Caldwell E., Peabody E. (2005). Incidence and outcomes of acute lung injury. *The New England Journal of Medicine*.

[2] Avecillas J. F., Freire A. X., Arroliga A. C. (2006). Clinical epidemiology of acute lung injury and acute respiratory distress syndrome: incidence, diagnosis, and outcomes. *Clinics in Chest Medicine*.

[3] Brower R. G., Matthay M. A., Morris A., Schoenfeld D., Thompson B. T., Wheeler A. (2000). Ventilation with lower tidal volumes as compared with traditional tidal volumes for acute lung injury and the acute respiratory distress syndrome. *The New England Journal of Medicine*.

[4] Villar J., Slutsky A. S. (2017). GOLDEN anniversary of the acute respiratory distress syndrome: still much work to do!. *Current Opinion in Critical Care*.

[5] Estenssoro E., Dubin A., Laffaire E. (2002). Incidence, clinical course, and outcome in 217 patients with acute respiratory distress syndrome. *Critical Care Medicine*.

[6] Brun-Buisson C., Minelli C., Bertolini G. (2004). Epidemiology and outcome of acute lung injury in European intensive care units. *Intensive Care Medicine*.

[7] Gong M. N., Thompson B. T., Williams P., Pothier L., Boyce P. D., Christiani D. C. (2005). Clinical predictors of and mortality in acute respiratory distress syndrome: potential role of red cell transfusion. *Critical Care Medicine*.

[8] Ware J. H. (2006). The limitations of risk factors as prognostic tools. *The New England Journal of Medicine*.

[9] Cooke C. R., Kahn J. M., Caldwell E. (2008). Predictors of hospital mortality in a population-based cohort of patients with acute lung injury. *Critical Care Medicine*.

[10] Wang C. Y., Calfee C. S., Paul D. W. (2014). One-year mortality and predictors of death among hospital survivors of acute respiratory distress syndrome. *Intensive Care Medicine*.

[11] Quesnel C., Piednoir P., Gelly J. (2012). Alveolar fibrocyte percentage is an independent predictor of poor outcome in Patients with acute lung injury. *Critical Care Medicine*.

[12] Blum J. M., Stentz M. J., Dechert R. (2013). Preoperative and intraoperative predictors of postoperative acute respiratory distress syndrome in a general surgical population. *Anesthesiology*.

[13] Walter J. M., Wilson J., Ware L. B. (2014). Biomarkers in acute respiratory distress syndrome: from pathobiology to improving patient care. *Expert Review of Respiratory Medicine*.

[14] Vajpayee N., Graham S. S., Bem S. (2011). *Basic Examination of Blood and Bone Marrow*.

[15] Mckenzie S. B., Williams J. L. (2004). *Clinical Laboratory Hematology*.

[16] Tonelli M., Sacks F., Arnold M., Moye L., Davis B., Pfeffer M. (2008). Relation between red blood cell distribution width and cardiovascular event rate in people with coronary disease. *Circulation*.

[17] Hu Z., Wei T., Tang Q. (2016). Prognostic value of red blood cell distribution width in acute pancreatitis patients admitted to intensive care units: an analysis of a publicly accessible clinical database MIMIC II. *Clinical Chemistry and Laboratory Medicine (CCLM)*.

[18] Huang Y. L., Hu Z. D., Liu S. J. (2014). Prognostic value of red blood cell distribution width for patients with heart failure: a systematic review and meta-analysis of cohort studies. *Plos One*.

[19] Osadnik T., Strzelczyk J., Hawranek M. (2013). Red cell distribution width is associated with long-term prognosis in patients with stable coronary artery disease. *BMC Cardiovascular Disorders*.

[20] Hu Y., Liu H., Fu S., Wan J., Li X. (2017). Red blood cell distribution width is an independent predictor of AKI and mortality in patients in the coronary care unit. *Kidney and Blood Pressure Research*.

[21] Braun E., Domany E., Kenig Y., Mazor Y., Makhoul B. F., Azzam Z. S. (2011). Elevated red cell distribution width predicts poor outcome in young patients with community acquired pneumonia. *Critical Care*.

[22] Xu H., Li W., Mao J., Pan Y. (2017). Association between red blood cell distribution width and Henoch–Schonlein purpura nephritis. *Medicine*.

[23] Bazick H. S., Chang D., Mahadevappa K., Gibbons F. K., Christopher K. B. (2011). Red cell distribution width and all-cause mortality in critically ill patients. *Critical Care Medicine*.

[24] Meynaar I. A., Knook A. H., Coolen S. (2013). Red cell distribution width as predictor for mortality in critically ill patients. *Netherlands Journal of Medicine*.

[25] Johnson A. E., Pollard T. J., Shen L. (2016). MIMIC-III, a freely accessible critical care database. *Scientific Data*.

[26] Wang B., Gong Y., Ying B., Cheng B. (2018). Association of initial serum total calcium concentration with mortality in critical illness. *BioMed Research International*.

[27] Amirian E. S., Zhou R., Wrensch M. R. (2016). Approaching a scientific consensus on the association between allergies and glioma risk: a report from the glioma international case-control study. *Cancer Epidemiology, Biomarkers & Prevention: A Publication of the American Association for Cancer Research, Cosponsored by the American Society of Preventive Oncology*.

[28] Gmallen F. (2007). Red cell distribution width as a novel prognostic marker in heart failure : data from the charm program and the duke databank. *Journal of the American College of Cardiology*.

[29] Bellani G., Laffey J. G., Pham T. (2016). Epidemiology, patterns of care, and mortality for patients with acute respiratory distress syndrome in intensive care units in 50 countries. *Journal of the American Medical Association*.

[30] Beal A. L., Cerra F. B. (1994). Multiple organ failure syndrome in the 1990s: systemic inflammatory response and organ dysfunction. *Journal of the American Medical Association*.

[31] Ware L. B., Eisner M. D., Thompson B. T., Parsons P. E., Matthay M. A. (2004). Significance of von Willebrand factor in septic and nonseptic patients with acute lung injury. *American Journal of Respiratory and Critical Care Medicine*.

[32] Ware L. B., Matthay M. A., Parsons P. E., Thompson B. T., Januzzi J. L., Eisner M. D (2007). Pathogenetic and prognostic significance of altered coagulation and fibrinolysis in acute lung injury/acute respiratory distress syndrome. *Critical Care Medicine*.

[33] Terpstra M. L., Aman J., van Nieuw Amerongen G. P., Groeneveld A. B. (2014). Plasma biomarkers for acute respiratory distress syndrome: a systematic review and meta-analysis. *Critical Care Medicine*.

[34] Lesur O., Langevin S., Berthiaume Y. (2006). Outcome value of Clara cell protein in serum of patients with acute respiratory distress syndrome. *Intensive Care Medicine*.

[35] Hu Z.-D., Chen Y., Zhang L. (2013). Red blood cell distribution width is a potential index to assess the disease activity of systemic lupus erythematosus. *Clinica Chimica Acta*.

[36] Kim J., Kim Y. D., Song T.-J. (2012). Red blood cell distribution width is associated with poor clinical outcome in acute cerebral infarction. *Thrombosis and Haemostasis*.

[37] Hu Z., Sun Y., Wang Q. (2013). Red blood cell distribution width is a potential prognostic index for liver disease. *Clinical Chemistry & Laboratory Medicine*.

[38] Montagnana M., Cervellin G., Meschi T., Lippi G. (2012). The role of red blood cell distribution width in cardiovascular and thrombotic disorders. *Clinical Chemistry and Laboratory Medicine*.

[39] Zhang Z., Xu X., Ni H., Deng H. (2013). Red cell distribution width is associated with hospital mortality in unselected critically ill patients. *Journal of Thoracic Disease*.

[40] Nathan S. D., Reffett T., Brown A. W. (2012). The red cell distribution width as a prognostic indicator in idiopathic pulmonary fibrosis. *Chest*.

[41] Silversides J. A., Ferguson A. J., McAuley D. F., Blackwood B., Marshall J. C., Fan E. (2015). Fluid strategies and outcomes in patients with acute respiratory distress syndrome, systemic inflammatory response syndrome and sepsis: a protocol for a systematic review and meta-analysis. *Systematic Reviews*.

[42] Huang X., Kong G., Li Y. (2016). Decitabine and 5-azacitidine both alleviate LPS induced ARDS through anti-inflammatory/antioxidant activity and protection of glycocalyx and inhibition of MAPK pathways in mice. *Biomedicine & Pharmacotherapy*.

[43] Brown S. M., Grissom C. K., Rondina M. T. (2015). Polymorphisms in key pulmonary inflammatory pathways and the development of acute respiratory distress syndrome. *Experimental Lung Research*.

[44] Ku N. S., Kim H.-W., Oh H. J. (2012). Red blood cell distribution width is an independent predictor of mortality in patients with gram-negative bacteremia. *Shock*.

[45] Ghaffari S. (2008). Oxidative stress in the regulation of normal and neoplastic hematopoiesis. *Antioxidants & Redox Signaling*.

